# Flint’s Medical Essays

**Published:** 1874-09

**Authors:** 


ESSAYS ON CONSERVATIVE MEDICINE AND KINDRED TOPICS. By Austin Flint, M.D., Professor of the Principles and Practice of Medicine, and of Clinical Medicine, in Bellevue Hospital Medical College, New York. Philadelphia: Henry C. Lea. 1874. 12mo. Cloth, pp. 214. Price $1.38.

Having occasion to visit a patient lately by rail, we put the little volume into our pocket, and finished it upon the route. We find it full to overflowing of that very uncommon form of common sense which Dr. Flint prefers to denominate *good sense*, and which is so very characteristic of the author. He treats, besides conservative medicine, medicine in the past, the present, and the future; alimentation in disease; tolerance of disease; agency of the mind in etiology, prophylaxis, and therapeutics; and Divine design as exemplified in the natural history of disease.

